# Grand Challenges in Reproductive Endocrinology

**DOI:** 10.3389/fendo.2016.00169

**Published:** 2017-02-08

**Authors:** Claus Yding Andersen

**Affiliations:** ^1^Laboratory of Reproductive Biology, The Juliane Marie Centre for Women, Children and Reproduction, Copenhagen University Hospital, Faculty of Health and Medical Sciences, University of Copenhagen, Copenhagen, Denmark

**Keywords:** reproductive endocrinology, sex steroids, gonadotropins, peptide growth factors, ovary, testes, follicles, seminiferous tubles

Reproduction is a fundamental aspect of life in all species. In a biological sense, it can be argued that reproduction is the essence of life. Passing on ones genes to the next generation and constantly optimizing the gene pool available for each individual and the species as a whole are vital for continuation and endurance of the species. Reproduction and hormones are intimately connected; thus, hormones are a prerequisite for reproduction and govern nearly all aspects of successful creation in both females and males. Therefore, to numerous scientists, reproductive endocrinology is one of the most fascinating disciplines in science, and it is a pleasure that now finally there is a scientific journal dedicated specifically to reproductive endocrinology.

Despite the lack of specific journals dedicated entirely to reproductive endocrinology up until now, this area has had a huge impact on society with first the development of female oral contraceptives around 50 years ago followed by the development of *in vitro* fertilization techniques to help childless couples in 1970–1980s.

Regardless of an enormous amount of literature on sex steroid hormones accumulated over the years, there are constantly new aspects of their function in the body and indeed in the reproductive organs. One aspect that we will hear more about in the coming years is the aging population, in which especially the majority of women will spend up to half of their life in menopause depletion of ovarian sex steroids (1). The consequences on general health including osteoporosis and cardiovascular disease are all aspects within the area of reproductive endocrinology and highlight the need for discussing hormone replacement therapy and/or alternative strategies. The male equivalent testosterone and the effects of testosterone especially in the aging male is also a matter of debate these years, and it is furthermore discussed how to measure testosterone in the clinically most meaningful way (2).

Right from the beginning of the IVF era, ovarian stimulation with exogenous FSH preparations has been an indispensable component of successful treatment and numerous studies have been conducted to test different compounds and their effect on the reproductive outcome. Although pregnancy rates of modern day IVF treatment and ART in general have plateaued during the last decade, the journey of optimizing ovarian stimulation is still far from being completed since ongoing pregnancy still occurs in only a fraction of treatment cycles. Whereas the follicular phase of the menstrual cycle has received intense interest from the very beginning, final maturation of the follicles in connection with ovulation induction has for many years been performed using a large bolus of hCG as a surrogate for the mid-cycle surge of gonadotropins. Recent years have shown that the hCG trigger indeed secures maturation of oocytes and stimulates corpora lutea during the first half of the luteal phase, but also carries the risk of inducing ovarian hyper stimulation syndrome (OHSS) (3). In order to circumvent the risk of OHSS, the agonist trigger was recently introduced, releasing an endogenous surge of gonadotropins with much less LH-like activity as compared to the hCG bolus trigger, more resembling the natural mid-cycle surge (4). This method can be used in women co-treated with a GnRH antagonist. The agonist trigger concept secures final maturation of the pre-ovulatory follicles equally well as compared to the hCG trigger, however, it requires additional luteal phase support in order to maintain similar reproductive outcomes (5). The GnRHa trigger concept is now gradually replacing the hCG trigger and has basically opened the luteal phase for further optimization (6). Presently, a number of studies are trying to introduce daily micro-dose hCG or rec-LH administration as a more appropriate luteal phase support than the standard vaginal support. The luteal phase support is the last frontier in ovarian stimulation, which needs to be carefully scrutinized, and it is still an open field that holds the promise of potentially optimizing results further. Importantly, the GnRHa trigger has already found its place in general practice in connection with patients at risk of OHSS development and in oocyte donors (4).

The development of multiple follicles *via* stimulation with exogenous FSH preparations has invariably been accompanied with supra-physiological levels of estradiol and progesterone as each follicle contributes to the overall concentration observed in circulation. Many effects of estradiol in the body are obviously reinforced by these high levels, including a risk of inducing a pituitary premature LH surge and also negative effects on the receptivity of the endometrium. Recent years have shown that the endometrium is inappropriately affected by high levels of estradiol and the progesterone during the early luteal phase. It has, somewhat surprising, now been shown that—if an efficient program for freezing oocytes and embryos is available—results of a freeze-all strategy with replacement in a subsequent natural cycle leads to higher reproductive outcomes, probably due to a more appropriate stimulation of the endometrium (7). This has raised questions on whether it is possible to reduce the output or the effect of estradiol during the follicular phase by for instance co-administrating aromatase inhibitors or estrogen receptor antagonists to avoid the estrogenic effect on the endometrium.

There is now also a move toward new strategies for ovarian stimulation, more in accordance with the gonadotropin actions of the natural cycle. One such option is to mimic the natural menstrual cycle and use either exogenous LH or hCG for stimulation of follicular growth in the second half of the follicular phase until ovulation induction where LH receptors are expressed on the granulosa cells and exogenous LH or hCG may substitute for exogenous FSH (8–10).

In the vast majority of cases, the pituitary is fully capable of producing all the FSH and LH that is required for stimulation of multiple follicular development—we need to be able to control its release, and a number of new combinations of old and new drugs are now becoming interesting and will probably be part of new stimulation regimes. It is also of interest to follow the development of new hormones for stimulation with a prolonged half-life and a pharmacokinetic profile that resembles more the release of the hormones in the natural cycle (6).

The apparently ever increasing maternal age of child bearing has become an important challenge for many fertility specialists, constituting an underlying important task in the management of the infertile patient. The development of appropriate hormonal tests to determine the biological age in a reproductive context and potential ovarian response (e.g., AMH, FSH, and inhibin-B) receives great attention and has proved clinically valuable. However, these tests are likely to be further improved during the coming years. Although the ovarian reserve in reproductive aged women is most often reduced, the number of resting follicles usually constitutes thousands of follicles. Focus is now turning toward this huge pool of early stage follicles, which during normal circumstances undergo atresia—maybe due to inappropriate hormonal stimulation rather than an in built attenuated developmental competence. Attempts to enhance utilization of this pool of early stage follicles to augment subsequent mature oocytes available for treatment are now under way. Alternatively, it may become possible to use part of an increased pool of oocytes to augment oocyte quality of other autologous oocytes. More specifically, we are now starting to understand the hormonal signaling pathways that result in initiation of growth in the pool of resting primordial follicles, i.e., activation of follicle growth (11–13). A new concept to augment the number of mature follicles is now being developed in which women with poor ovarian response have a biopsy of the ovary, which is subsequently subjected to activation of the pool of resting follicles during a short *in vitro* procedure after which the tissue is replaced again. During the next months, the pool of activated follicles will grow to the pre-ovulatory stage. In the best case scenario, more mature follicles will result. Ovarian stimulation for the last 2 weeks with exogenous hormones is likely to be part of achieving full maturity of these follicles. Furthermore, understanding the hormonal regulation of follicle growth will be a key feature in developing such concepts to a more widespread clinical level, hopefully backed by studies in rodents, large domestic species, and primates.

Overall, we are still only starting to understand the regulation of follicular growth and indeed how the highly specialized follicular compartment is hormonally regulated (14). Numerous studies have been performed on human follicular fluid from fully mature follicles obtained in connection with retrieval of oocytes during IVF treatment. This follicular fluid represents, however, the final stage of follicular development, in which the follicle and the surrounding theca cells are turning into a corpus luteum that will function during the luteal phase. At this time point, the cells are undergoing dramatic changes including alterations in gonadotropin receptor expression. Earlier stages of follicular development have been studied only to a limited extend, especially because human material is very limited for obvious reasons and because animal models often present with small sized follicles with a limited number of cells making detailed studies difficult. However, important information will continue to be obtained from animal models and especially large domestic species, for instance the bovine. A better understanding of the milieu inside small antral follicles and their response to hormones derived outside and/or inside the follicles is important basic science information, which will help optimize conditions for *in vitro* maturation of oocytes and potentially develop new therapeutic approaches that can replace the existing IVF procedures. The long-term goal is to be able to grow follicles from the earliest stages of development and derive mature follicles capable of sustaining fertilization resulting in healthy offspring. This has been demonstrated in rodents; however, a huge research effort is required to take this step in humans.

After the development of the ICSI procedure, endocrinology in males received less attention for some years, but new information on how to improve sperm function and the hormonal regulation of testicular function is now appearing. It appears that some men with poor sperm quality may benefit from exogenous FSH stimulation (15). The importance of specific FSH isoforms and genetic variants and FSH receptor polymorphisms (i.e., single nucleotide polymorphisms) are being evaluated and a potential sensitizing effect of co-administration of LH-like activity (e.g., hCG) is another option to potentially improve sperm production. The identification of subgroups of patients with a specific combination of SNP’s that is more likely to benefit from this treatment is a possible scenario that is being tested. The effects of testosterone and especially low concentrations of testosterone also receives interest and the debate on whether to augment concentrations therapeutically is a hot issue—just highlighting that more research and information is required to make informed decisions.

Studies in animals have been and will continue to be a cornerstone in understanding reproductive endocrinology. The creation of genetically manipulated mice—for instance, mice with a gene globally knocked out—and more recently conditional knock out models have provided valuable information. However, mice are not humans and significant differences exist. Primate or human studies are required to confirm the information from animal studies. One example is AMH, which in mice appears to attenuate follicle recruitment from the resting stage (16). Recent studies now suggest that the opposite may be the case in women, where two independent studies now have shown that AMH appears to stimulate follicular recruitment (17, 18). This may reflect that regulation of the biological activity and proteolytic cleavage of AMH is complicated; it may differ between sexes and is far from being elucidated (19). Later on in folliculogenesis, AMH has a number of different functions—one of which is to reduce estradiol output by small antral follicles before they become selected and chosen to undergo ovulation (20). AMH belongs to the group of TGF-β growth factors, which have been shown to undertake a huge number of hormonal functions in the gonads. We are far from understanding all of the functions of these growth factors, their fine tuning, regulation of biological activity, and interplay with other hormones. The presence of functional homodimers and heterodimers between different members of the TGF-β family is new and the functional significance on both the ovary and testes remains to be determined (21).

The ability to successfully freeze both human ovarian and testicular tissue has allowed development of new treatments to preserve fertility by excising and freezing tissue prior to gonadotoxic treatment often in connection with a cancer disease. The transplantation of the tissue to either the remaining ovary or outside the ovary creates situations, which have not been seen previously. The development of follicles, their hormonal secretion, and the hormonal control of follicular growth provide an opportunity to study different conditions that can highlight other sides of reproductive endocrinology. The ability to freeze ovarian tissue and the enormous number of resting follicles in the ovaries, which to a large extent maintain the capacity to produce female sex steroid, has also let to the proposal of more futuristic approaches—it is now technically possible to postpone menopause by freezing ovarian tissue during the reproductive years and only replace it after menopause (22, 23). Importantly, it will not harm the woman—the fertility and the age at menopause do not seem to be significantly negatively affected—and the increased utilization of the woman’s own follicles will lead to circulating levels of estradiol and progesterone, which will effectively prevent osteoporosis and cardiovascular disease. However, continued menstrual cycling may lead to unwanted side effects such as an increased risk of breast cancer, and benefits and drawbacks will need to be evaluated carefully in the coming years.

Spermatogonia of intact human testicular tissue are also capable of surviving freezing and the tissue maintains the capacity to secrete specific hormones like AMH and testosterone. However, the specific hormonal requirements to culture spermatogonia *in vitro* are not yet in place, and although animal models are not similar to the human situation, more information is required to understand the physiology better and to potentially reach an expertise allowing a clinical approach.

Taken together, reproduction is still a very research intensive area with many different disciplines, which calls for a multitude of different approaches to obtain a better understanding of the physiology of producing new offspring and expand on the endocrinology that governs fertility. It is the aim that this new initiative—the Reproduction section of *Frontiers*—will become an integrated part of advancing this field in science.

## Author Contributions

CA wrote the manuscript, and views and opinions expressed only represent those of the author.

## Conflict of Interest Statement

The author confirms being the sole contributor of this work and approved it for publication.
